# Community‐based wildlife management area supports similar mammal species richness and densities compared to a national park

**DOI:** 10.1002/ece3.5916

**Published:** 2019-12-06

**Authors:** Christian Kiffner, Seth Thomas, Talia Speaker, Victoria O'Connor, Paige Schwarz, John Kioko, Bernard Kissui

**Affiliations:** ^1^ Center for Wildlife Management Studies The School For Field Studies Karatu Tanzania; ^2^ Department of Integrative Biology & The Department of Environmental Sciences Oregon State University Corvallis OR USA; ^3^ Human Dimensions of Natural Resources Colorado State University Fort Collins CO USA; ^4^ Department of Psychology Franklin & Marshall College Lancaster PA USA; ^5^ Warner College of Natural Resources Colorado State University Fort Collins CO USA

**Keywords:** community‐based conservation, conservation effectiveness, distance sampling, ecological baseline, participatory monitoring, population dynamics

## Abstract

Community‐based conservation models have been widely implemented across Africa to improve wildlife conservation and livelihoods of rural communities. In Tanzania, communities can set aside land and formally register it as Wildlife Management Area (WMA), which allows them to generate revenue via consumptive or nonconsumptive utilization of wildlife. The key, yet often untested, assumption of this model is that economic benefits accrued from wildlife motivate sustainable management of wildlife. To test the ecological effectiveness (here defined as persistence of wildlife populations) of Burunge Wildlife Management Area (BWMA), we employed a participatory monitoring approach involving WMA personnel. At intermittent intervals between 2011 and 2018, we estimated mammal species richness and population densities of ten mammal species (African elephant, giraffe, buffalo, zebra, wildebeest, waterbuck, warthog, impala, Kirk's dik‐dik, and vervet monkey) along line transects. We compared mammal species accumulation curves and density estimates with those of time‐matched road transect surveys conducted in adjacent Tarangire National Park (TNP). Mammal species richness estimates were similar in both areas, yet observed species richness per transect was greater in TNP compared to BWMA. Species‐specific density estimates of time‐matched surveys were mostly not significantly different between BWMA and TNP, but elephants occasionally reached greater densities in TNP compared to BWMA. In BWMA, elephant, wildebeest, and impala populations showed significant increases from 2011 to 2018. These results suggest that community‐based conservation models can support mammal communities and densities that are similar to national park baselines. In light of the ecological success of this case study, we emphasize the need for continued efforts to ensure that the BWMA is effective. This will require adaptive management to counteract potential negative repercussions of wildlife populations on peoples' livelihoods. This study can be used as a model to evaluate the effectiveness of wildlife management areas across Tanzania.

## Graphical abstract

Our case study uses time‐matched and consecutive wildlife counts over multiple years in a community‐based conservation scheme and in an ideal baseline (a directly neighboring national park) as comparison. We show that mammal species richness and densities of ten mammal species are not fundamentally different in a community‐based conservation area versus the adjacent national park. These results showcase the high conservation value of this community‐based conservation model.

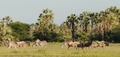

## 1 INTRODUCTION

Community‐based natural resource management models have been advocated as a dual strategy to alleviate poverty and to halt overall biodiversity decline (Berkes, 2004, 2007; Kiss, 1990, 2004). In East Africa, where wildlife populations have been declining (Craigie et al., 2010; Stoner et al., 2007; Western, Russell, & Cuthil, 2009) and people in rural areas often lack basic commodities (Ellis & Freeman, 2004; Reardon & Vosti, 1995; Salerno, Borgerhoff Mulder, Grote, Ghiselli, & Packer, 2016), community‐based wildlife conservation models have been considered and implemented as a strategy to balance the trade‐off between wildlife conservation and development (Borgerhoff Mulder & Coppolillo, 2005; Kiwango, Komakech, Tarimo, & Martz, 2015; Naidoo et al., 2016). In East Africa and elsewhere, such community‐based conservation models have been subject to considerable criticism, specifically in regard to their socio‐economic contributions and poor governance (Benjaminsen, Goldman, Minwary, & Maganga, 2013; Bluwstein, Moyo, & Kicheleri, 2016; Brehony, Bluwstein, Lund, & Tyrrell, 2018; Goldman, 2003; Moyo, Ijumba, & Lund, 2016; Wright, 1995). While constructive criticism may improve issues related to benefit sharing and local involvement in governance over natural resources, research on the ecological effectiveness of community‐based conservation models is an equally important component to guide adaptive management and policy (Lee & Bond, 2018; Lindenmayer & Likens, 2010; Ogutu, Kuloba, Piepho, & Kanga, 2017; Stem, Margoluis, Salafsky, & Brown, 2005; Watson, Dudley, Segan, & Hockings, 2014; Yoccoz, Nichols, & Boulinier, 2001). Exemplary work in multipurpose and community‐based conservation areas in East Africa suggests that these areas can support species‐rich and abundant wildlife communities (Georgiadis, Olwero, Ojwang', & Romañach, 2007; Kinnaird & O'Brien, 2012; Schuette, Creel, & Christianson, 2016), yet long‐term wildlife monitoring in these areas is often lacking (Newmark & Hough, 2000).

Assessing the performance of conservation areas requires monitoring of suitable biological variables over time, ideally in comparison to appropriate spatial baselines (Geldmann et al., 2015; Schmeller et al., 2018). When wildlife is readily observable, assessing, and estimating species richness of large mammal assemblages and population densities of specific species over time can be performed simultaneously (Kiffner et al., 2019; Kiffner, Nagar, Kollmar, & Kioko, 2016; Schuette et al., 2018). This combined approach offers advantages over focusing solely on species richness (Cromsigt, van Rensburg, Etienne, & Olff, 2009; Msuha, Carbone, Pettorelli, & Durant, 2012; Treydte, Edwards, & Suter, 2005), on one or few snapshot assessments of species' densities (Caro, 1999; Waltert, Meyer, & Kiffner, 2009), or on population trends of selected species (Kiffner et al., 2017; Ogutu et al., 2017). This is because (a) mammal communities are sensitive to different levels of human impact (Kiffner, Wenner, LaViolet, Yeh, & Kioko, 2015; Msuha et al., 2012; Riggio et al., 2018); (b) focusing on one snapshot assessment in time may yield biased conclusions if animals move across the landscape in response to seasonal variation of natural resources (Rannestad, Danielsen, & Stokke, 2006); and (c) focusing on a single species may not represent population trajectories of other species (Caro, 2016; Caro, Gardner, Stoner, Fitzherbert, & Davenport, 2009; Kiffner, Hopper, & Kioko, 2016; Riggio et al., 2018).

Here, we report on a participatory (i.e., involving local management personnel) monitoring study (Danielsen, Burgess, & Balmford, 2005; Msoffe et al., 2010) to estimate mammalian species richness and densities of ten mammal species in Burunge Wildlife Management Area, Tanzania. In contrast to other assessments of the effectiveness of wildlife management areas in northern Tanzania, which used animal population metrics on community lands as baseline for comparisons (Lee, 2018; Lee & Bond, 2018), we chose to compare wildlife community and population parameters to those assessed in an adjacent fully protected national park (Tarangire National Park). Using a national park as baseline allowed us to assess whether wildlife management areas can support the same structure and abundance of wildlife populations compared to areas where human influence is minimal (Arcese & Sinclair, 1997; Sinclair & Dobson, 2015).

## 2 MATERIAL AND METHODS

### 2.1 Study areas

**Figure 1:**
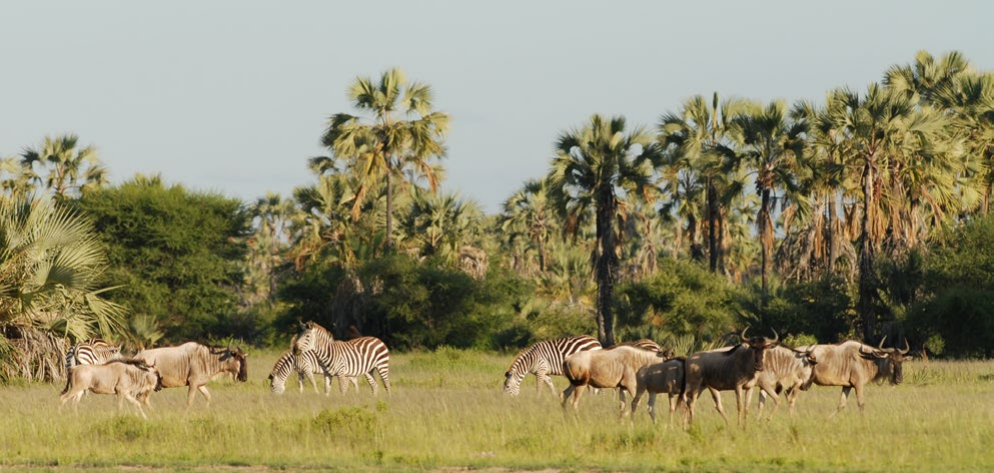
Wildebeest (*Connochaetes taurinus*) and zebra (*Equus quagga*) in Burunge Wildlife Management Area, Tanzania (Photo: C. Kiffner)

**Figure 2:**
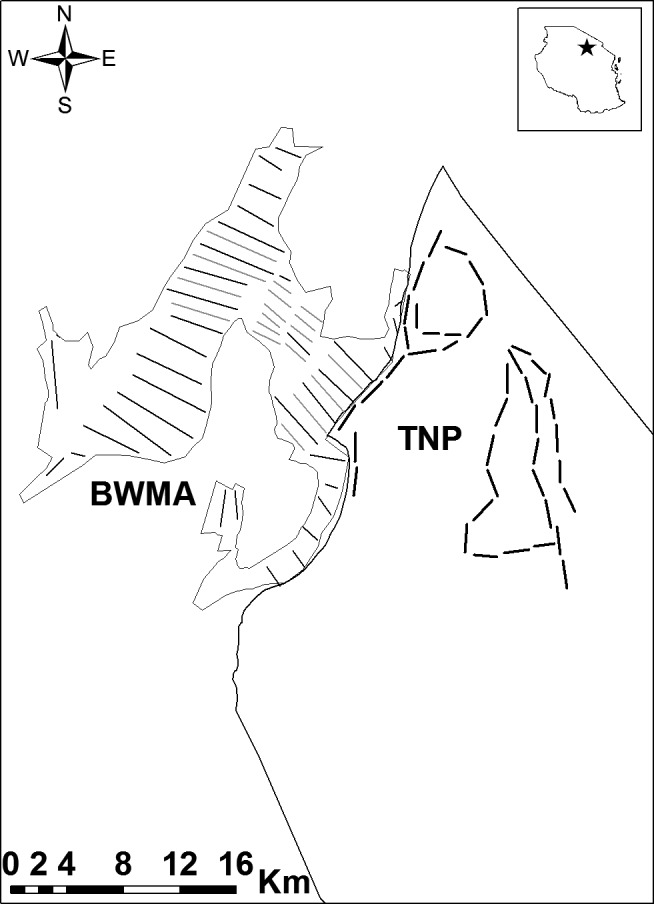
Map of the study area, showing the outlines of Tarangire National Park (TNP), the wildlife area of Burunge Wildlife Management Area (BWMA) and the transects used for animal counts. Additional transects during the 2018 BWMA survey are displayed in light gray. The inset (top right) indicates the approximate location of the study area within Tanzania

Burunge WMA (BWMA) and Tarangire NP (TNP) are located in the Tarangire‐Manyara Ecosystem (TME). The TME is characterized by the movements of wildebeest (*Connochates taurinus*) and zebra (*Equus quagga*) populations (Figure 1) which perform round‐trip migrations to track seasonal landscape‐scale variation in food and nutrient availability (Bolger, Newmark, Morrison, & Doak, 2008). The climate of the area is semiarid, with an average annual rainfall of 710 mm (Prins & Loth, 1988). The topography is flat to undulating, and the average elevation is between 1,000 m (BWMA) and 1,200 m (TNP) above sea level (Gereta, Ole Meing'ataki, Mduma, & Wolanski, 2004; Lee, 2018). The landscape is characterized by mosaics of *Acacia‐Commiphora* bushland, edaphic grasslands, and riverine vegetation (Lamprey, 1964; Prins & Loth, 1988). BWMA and TNP share a common, unfenced border (Figure 2).

BWMA, officially gazetted in 2006, is located in the Babati district, contains ten villages, and the main ethnic groups are pastoralist Maasai and agro‐pastoral Mbugwe (Kaswamila, 2012). In Tanzania, wildlife management areas are spatially structured by land‐use plans that assign specific human activities to designated areas. The portion of BWMA that is set aside for wildlife use only is 280 km^2^ in extent (226 km^2^ excluding Lake Burunge). Initially, the eastern portion of the wildlife area was designated for trophy hunting, but since 2014 only photographic tourism has been practiced in the wildlife area (Lee, 2018). Village game scouts regularly patrol the area by vehicle and on foot to mitigate illegal activities such as livestock grazing, tree cutting, and hunting. Due to support from multiple donors, the management of the BWMA and law enforcement activities have been substantially strengthened since 2015 (Lee, 2018).

Tarangire National Park is 2,850 km^2^ in extent, but animal counts for this study were restricted to the northern section of the park (approx. 300 km^2^) that directly borders BWMA. This sector of TNP contains exceptionally high wildlife densities during the dry season (June–November) due to the availability of water and grass during this time (Kiffner, Hopper, et al., 2016; Lamprey, 1964). At the start of the rainy season (usually November), multiple species (mainly wildebeest and zebra) leave TNP and migrate to the Simanjiro plains in the east or to the northern plains around Lake Natron (Bond, Bradley, Kiffner, Morrison, & Lee, 2017; Borner, 1985; Lamprey, 1964) where they give birth in nutrient‐rich grasslands (Morrison & Bolger, 2012; Voeten, van de Vijver, Olff, & van Langevelde, 2009). At the onset of the dry season (usually around June), wildebeest and zebra return to TNP. TNP is managed by Tanzanian National Parks (TANAPA), whose rangers regularly patrol the area. Other than photographic tourism and research, no human activities are permitted.

### 2.2 Wildlife surveys

We used ground‐based line transect methodology to estimate large mammal presence and density in the northern sector of TNP and the wildlife use area of BWMA (Buckland et al., 2001; Thomas et al., 2010). To account for the seasonal distribution of wildlife in the TME, we collected line transect data during varying seasons from 2011 to 2018.

With a few exceptions, we placed transects in BWMA systematically and oriented them parallel to each other if landscape features allowed (Figure 2). Overall, we completed seven separate surveys in BWMA. Transects varied in length (range: 0.5–10.3 km, average: 3.4 km), and for the most part, we completed the same transects in each survey. In 2018, we added multiple new transects to increase the spatial coverage of the area. In total, we counted wildlife along 871.33 km of transects. We performed animal counts with teams of 3–7 observers per transect including BWMA game scouts, wildlife division rangers, and students from the School for Field Studies (2014 survey only) and used GPS units with preloaded transect coordinates (Garmin Etrex & GPS map 760Cx) to navigate in the field. We surveyed wildlife by either walking or driving with a 4WD vehicle along transects. We walked transects in the dense bushlands near the border with TNP and drove transects located in more open areas in the grasslands near the shore of Lake Manyara (Figure 2). For each animal sighting, we recorded the species, group size (defined as individuals of the same species within 50 m), and GPS coordinates. We measured perpendicular distances between the center of the animal (group) and the transect directly in the field with Bushnell Elite 1500 laser range finders. Observers walked along the transect until reaching orthogonal bearings with the animal (group). If animals moved after the initial detection, observers recorded the perpendicular distance between the transect line and the initial position of the animal (group). Prior to the start of each survey, we trained all participating individuals on data collection and equipment use.

Due to off‐road restrictions in TNP, we placed transects along major roadways within the park. To distribute transects as representative as possible, we chose roads that cover all major habitats (bushed grasslands in the north, *Vachellia tortilis* savanna in the central part, riverine habitat near the Tarangire river and *Combretum‐Terminalia* shrubland at higher elevations) of the northern section of TNP (Figure 2). Transects were generally 2 km in length (range: 0.3–2.5 km, average: 2 km), consecutive transects and were separated by 500 m. In total, we conducted six surveys in TNP, totaling 484.05 km. We drove transects in open‐top vehicles at slow speed, and 3–9 observers recorded mammal sightings. We followed the same protocol as in BWMA to assess, count, and record animal sightings. We used the same GPS units and range finders and trained all observers in data collection prior to fieldwork.

We aimed to time‐match surveys in BWMA and TNP as closely as possible to avoid potential confounding effects of seasonal animal movements. Time intervals between matched surveys typically ranged between 11 and 14 days. The 2018 dry season surveys were an exception at 40 days apart, but wildlife movement in this ecosystem is limited during the dry season (Gereta et al., 2004; Lamprey, 1964; Morrison, Link, Newmark, Foley, & Bolger, 2016). The first survey in BWMA (2011 long rain) had no equivalent TNP survey but we included density estimates from this survey to assess annual trends of wildlife populations in BWMA.

### 2.3 Data analyses

To generate seasonal species richness estimates for each study area, we used the program *EstimateS 9* (Colwell, 2016). We estimated the expected number of species as a function of sampling effort (number of transects). We derived rarefaction curves based on recorded (naive) occurrence data of each mammal species and 100 bootstrap replicates. In contrast to species richness estimation in a multispecies occupancy framework (which typically requires repeat visits in the same season and allows incorporating variable detectability of species), estimates from this method may be considered minimum estimates of species richness (Mc New & Handel, 2015).

Genet (*Genetta genetta* and *G. tigrina*) and mongoose (*Helogale* spp., *Herpestes* spp. & *Mungos* spp.) species were not always unambiguously identified to species level and were thus combined to “genet” or “mongoose” (Appendix S1). We visually compared species richness estimates at the community‐level. In addition, since transect length varied systematically between BWMA and TNP, we compared species richness estimates at the transect level using a generalized linear model (glm) with Poisson error distribution. This error distribution was selected because species richness is a count metric and was not normally distributed (Shapiro–Wilk test: *W* = 0.93, *p* < .001). We tested for area and seasonal effects on species richness estimates and included transect length as an explanatory variable to account for differing transect length. We first fitted the most complex model (area + season + transect length) and then derived all permutations of variable combinations using the *MuMIn* package (Bárton, 2013). Because the top two models received similar information‐theoretic support (within two AICc scores), we averaged these models using the full average method (Burnham & Anderson, 2002; Grueber, Nakagawa, Laws, & Jamieson, 2011).

We estimated species‐ and area‐specific detection functions with sighting data from the most frequently detected mammal species using Distance 6.0 (Thomas et al., 2010). To estimate densities for a range of species, we considered species with a minimum of 20 sightings. However, 13 out of the 20 species‐area combinations yielded more than the recommended 60 sightings (Buckland et al., 2001). For each species–area combination, we fitted half‐normal detection functions [which is the recommended shape of the detection function, particularly with limited detection frequencies (Prieto Gonzalez, Thomas, & Marques, 2017)] in the conventional (CDS) and the multicovariate (MCDS, with season coded as a three‐level covariate) distance sampling framework. We selected between CDS and MCDS models based on Akaike's information criterion. For all models, we truncated 10% of the observations most distant from transects (Buckland et al., 2001) and used the average cluster size in each season to extrapolate from herd density to animal density (ind./km^2^).

We compared density estimates using a pairwise testing approach because density estimates in BWMA and TNP were derived from time‐matched surveys. For each species–season combination, we tested for significant density differences using a *z* test (Buckland et al., 2001). Due to multiple testing (*n* = 6 pairwise comparisons for each species), we corrected the corresponding *p*‐value using the Bonferroni method (Kiffner, Nagar, et al., 2016). To assess temporal trends in the time series of the population density estimates, we computed Kendall's correlation test. All statistics and graphs were computed and generated in *R 3.6* (R Core Team, 2016).

## 3 RESULTS

### 3.1 Large mammal species richness

**Figure 3:**
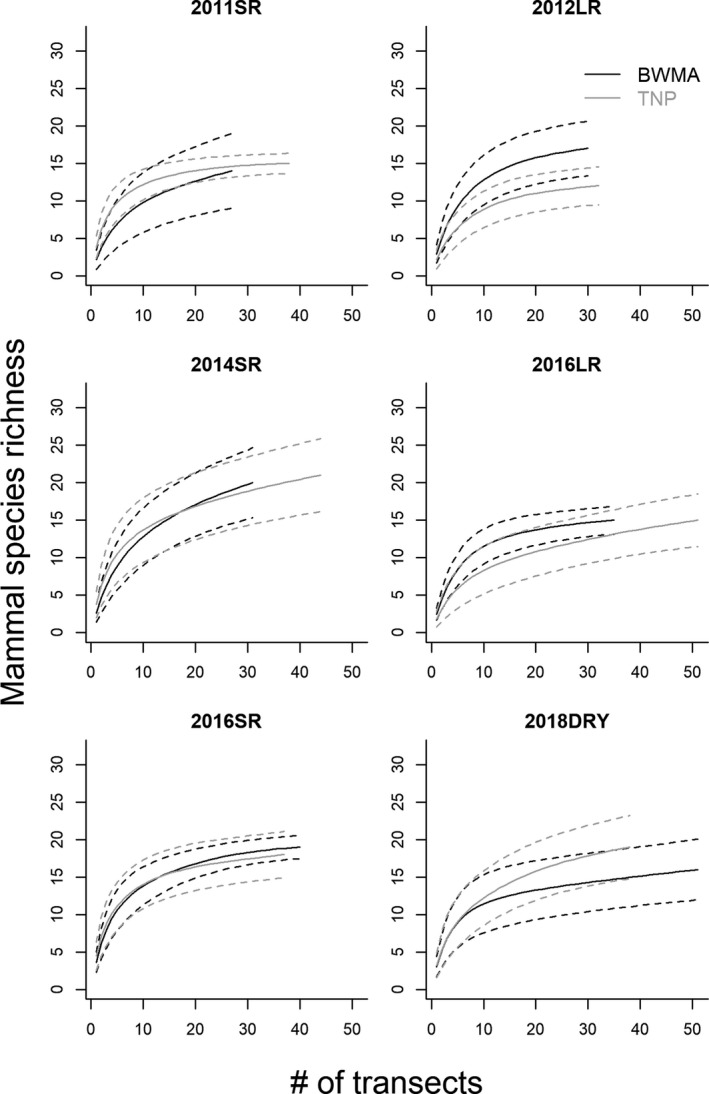
Species richness estimates of seasonal (SR: short rain season; LR: long rain season; Dry: dry season) large mammal species richness in Burunge Wildlife Management Area (BWMA; black) and Tarangire National Park (TNP; gray). Solid lines represent mean estimates; dashed lines indicate 95% confidence intervals of the mean estimates

Season‐ and site‐specific species accumulation curves appeared to be asymptotic, suggesting that sampling effort was sufficient (Figure 3). In general, both study areas appeared to support similar mammal species richness (Figure 3) and there was a high congruence in observed mammal species between BWMA and TNP. However, klipspringer (*Oreotragus oreotragus*) and bush hyrax (*Heterohyrax brucei*) were only observed in TNP, while hippopotamus (*Hippopotamus amphibius*), genets, hares (*Lepus* spp.), spotted hyenas (*Crocuta crocuta*), leopards (*Panthera pardus*), and African wild cats (*Felis lybica*) were only recorded in BWMA (Appendix S1). At the transect level, however, observed mammal species richness was—on average and corrected for variable transect length—higher in TNP compared to BWMA and was lower during the long rainy season compared to surveys during the short rains (Appendix S2).

### 3.2 Seasonal population densities and population trends over time

**Figure 4:**
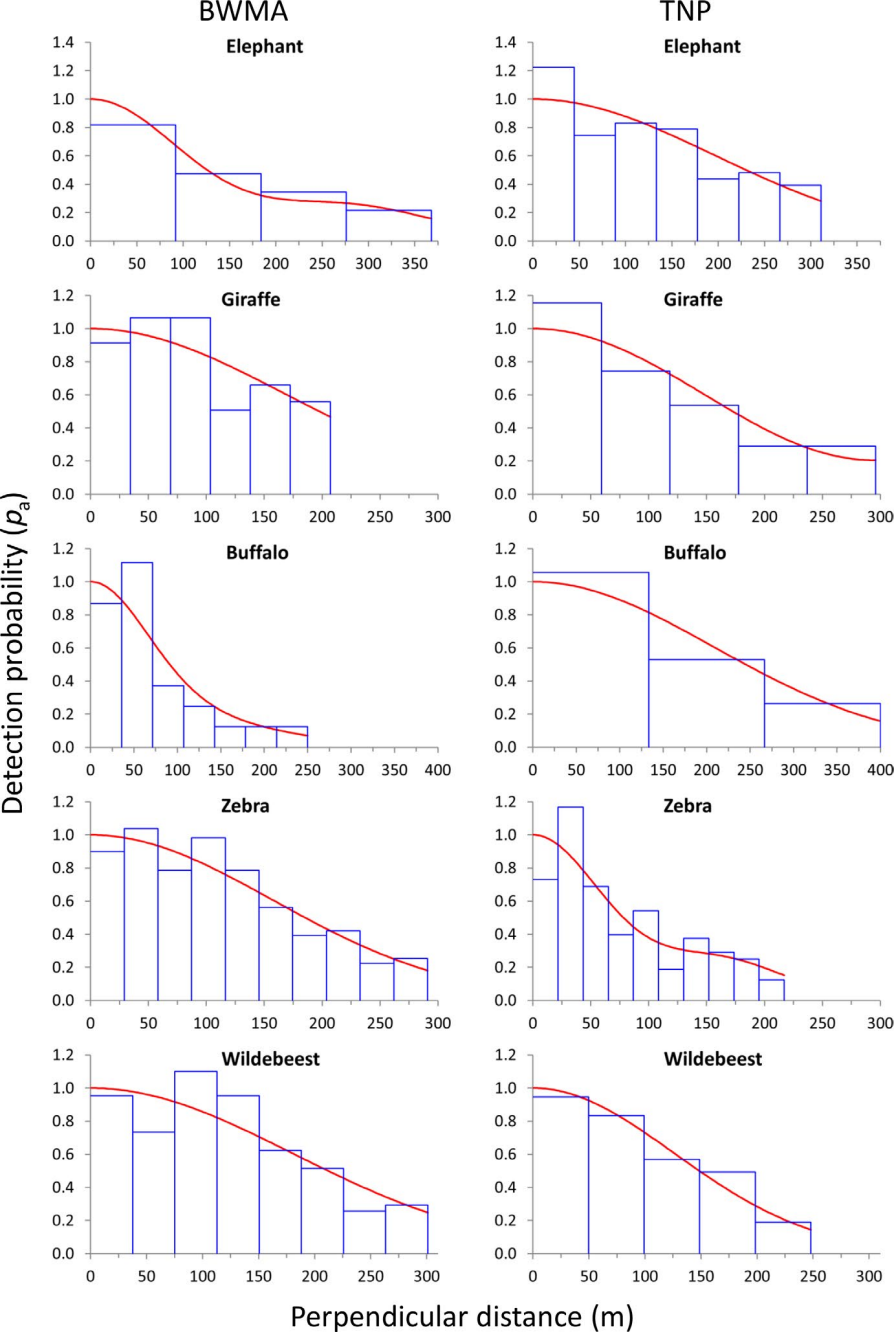
Detection functions of elephant, giraffe, buffalo, zebra, and wildebeest in Burunge Wildlife Management Area (BWMA) and Tarangire National Park (TNP). Histograms (blue bars) represent sighting frequencies and the red line describes the fitted detection function

**Figure 5:**
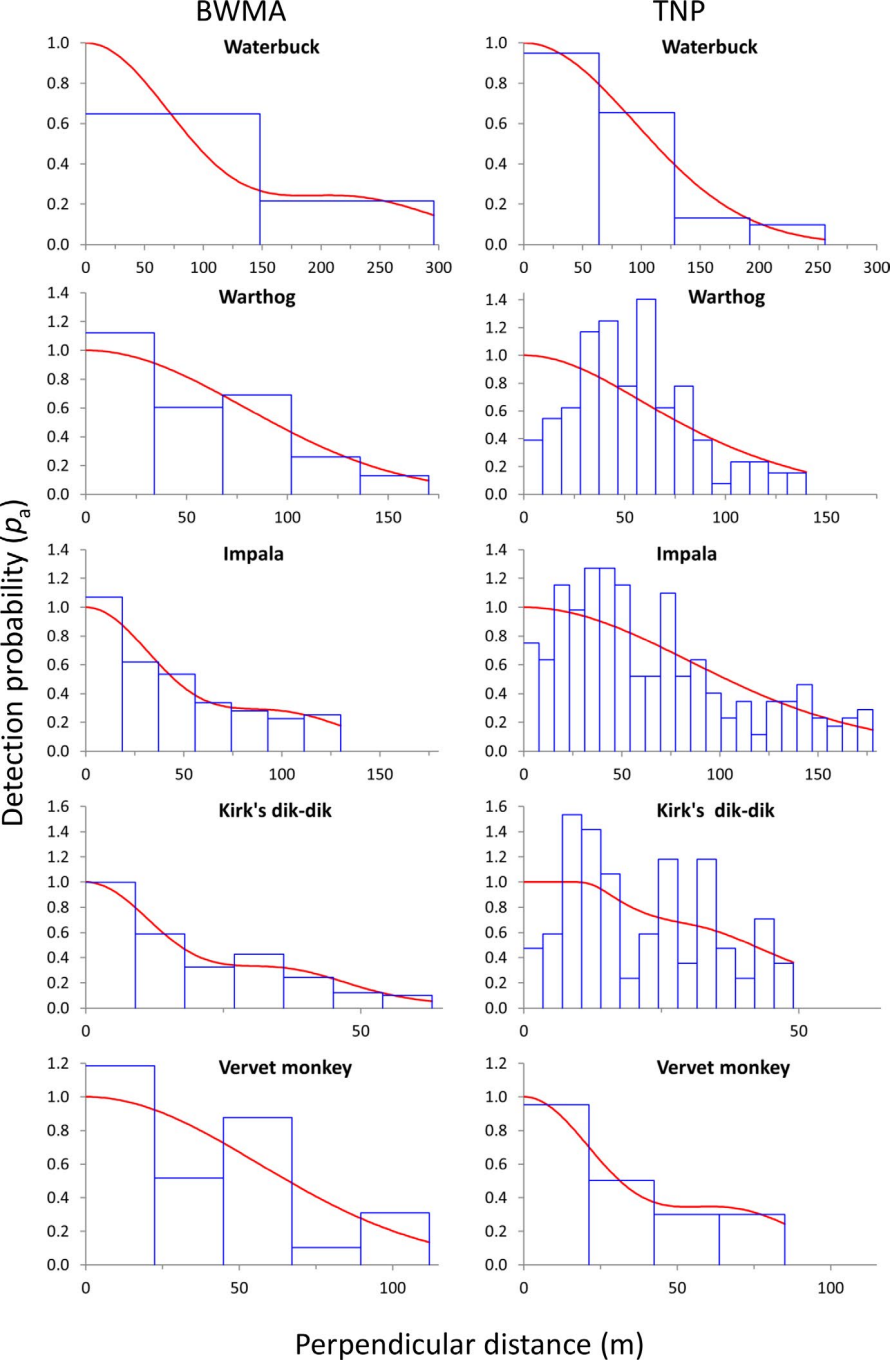
Detection functions of waterbuck, warthog, impala, Kirk's dik‐dik, and vervet monkey in Burunge Wildlife Management Area (BWMA) and Tarangire National Park (TNP). Histograms (blue bars) represent sighting frequencies and the red line describes the fitted detection function

In most of the species–site combinations (16 out of 20), CDS half‐normal detection functions were selected, but in four cases (BWMA: buffalo; TNP: warthog, impala, Kirk's dik‐dik) the MCDS half‐normal detection function better fit the data (Appendix S3). Mostly, the selected detection functions fit the observed data as indicated by chi^2^‐goodness of fit *p*‐values exceeding .05 (except for two cases BWMA: wildebeest; TNP: warthogs) (Appendix S2). However, some species appear to avoid areas near the transect line as indicated by fewer observations in the first distance bin(s) (Figures 4 and 5). This pattern was particularly observed in road transects in TNP (zebra, warthog, impala, and Kirk's dik‐dik) and much less pronounced in data from the walking transects in BWMA (Figures 4 and 5).

**Figure 6:**
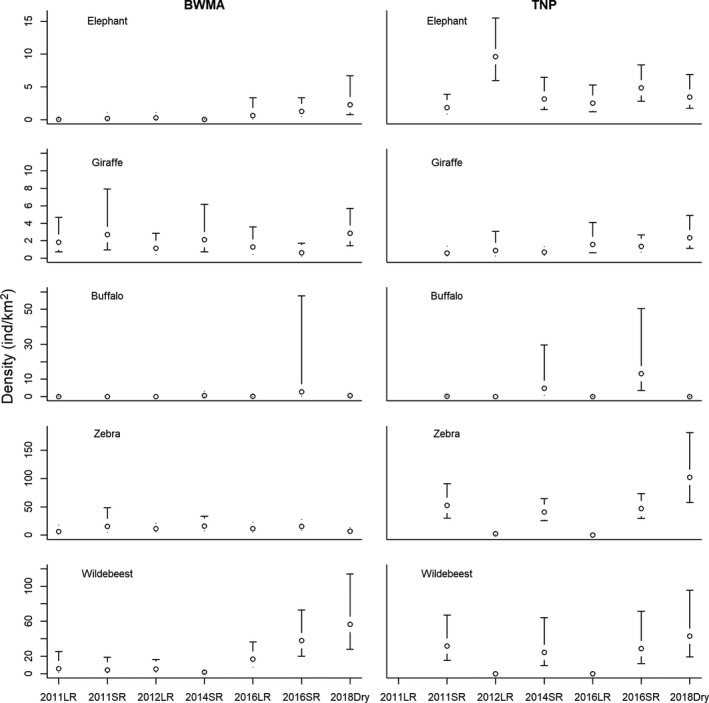
Estimated seasonal (SR: short rain season; LR: long rain season; Dry: dry season) densities (open circles) and associated 95%‐confidence intervals (error bars) of waterbuck, warthog, impala, Kirk's dik‐dik, and vervet monkey in Burunge Wildlife Management Area (BWMA) and Tarangire National Park (TNP)

**Figure 7:**
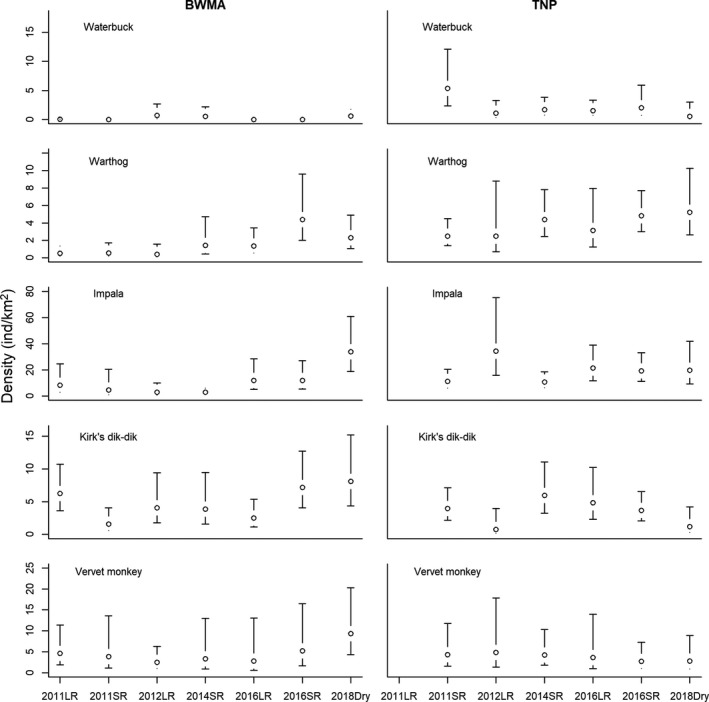
Estimated seasonal (SR: short rain season; LR: long rain season; Dry: dry season) densities (open circles) and associated 95%‐confidence intervals (error bars) of elephant, giraffe, buffalo, zebra, and wildebeest in Burunge Wildlife Management Area (BWMA) and Tarangire National Park (TNP)

Densities of larger‐ and smaller‐bodied species are displayed in Figures 6 and 7. Among the analyzed species, zebra, wildebeest, and impala occurred at high densities in both BWMA and TNP. Waterbuck, buffalo, and giraffe occurred at relatively low densities in both areas. Zebra and wildebeest population densities showed strong seasonal variability in TNP but not BWMA, with both species practically absent from TNP during the long rains but occurring at high densities during dry and short rain seasons.

Pairwise comparisons of seasonal densities indicated that species' densities in BWMA and TNP were not significantly different during most seasons (Appendix S4). However, in two out of six pairwise comparisons, elephants had significantly greater densities (long rains 2012 and short rains 2014) in TNP than in BWMA. In two out of six pairwise comparisons (short rains 2016 and dry season 2018) zebras also had significantly greater densities in TNP. In contrast, during the 2016 long rains, zebra density was significantly greater in BWMA than in TNP (Appendix S4).

Over the intermittent time series from 2011 to 2018, elephant, wildebeest, and impala populations showed a significant population increase in BWMA (Appendix S5). Concurrently, populations of giraffe and warthog showed a significant and positive trajectory in TNP, while vervet monkey populations declined over time.

## 4 DISCUSSION

The results of wildlife surveys in BWMA highlight that this community‐based conservation model (a) maintains a near‐complete mammal community, (b) supports high densities of resident and migratory large mammal species, and (c) effectively maintains populations of these species over time. These results thus confirm and bolster conclusions of previous studies in the Tarangire ecosystem, which suggest, that wildlife management areas in Tanzania can be ecologically effective (Lee, 2018; Lee & Bond, 2018).

### 4.1 Conservation value and implications of effective wildlife conservation

Mammal species richness, community composition, and densities similar to those observed in neighboring TNP emphasize the considerable conservation value of BWMA. Given the overall positive relationship between species richness and ecosystem functioning (Loreau, 2009, 2010), it can be expected that BWMA effectively supports crucial ecological processes in the wider landscape. BWMA provides suitable habitat for migratory ungulates (e.g., wildebeest and zebra) and nonmigratory species (e.g., Kirk's dik‐dik, impala, and giraffe) across seasons. Therefore, it is likely that BWMA not only helps to maintain connectivity within the increasingly fragmented Tarangire‐Manyara ecosystem (Bond et al., 2017; Borner, 1985; Morrison & Bolger, 2014; Morrison et al., 2016) but also provides year‐round habitat for numerous species. Indeed, BWMA supports populations of ungulate species (e.g., eland, hartebeest, lesser kudu, steenbuck, bush duiker, reedbuck) that are either rare or absent in other parts of the Tarangire‐Manyara ecosystem such as Lake Manyara National Park (Kiffner et al., 2015). Similar to other terrestrial protected area networks, BWMA thus likely provides complementary conservation functions in the wider Tarangire‐Manyara ecosystem which cannot be achieved by the current national park network alone (Caro et al., 2009; Gardner et al., 2008; Leménager, King, Elliott, Gibbons, & King, 2014).

Species accumulation curves in TNP occasionally did not fully reach asymptotes (Figure 3) which may suggest that mammal species richness in TNP may be slightly greater than in BWMA. In line with this finding, species richness per transect in BWMA was lower than in TNP. Slightly lower species richness can potentially be explained by multiple hypotheses. In BWMA, transects occasionally covered only a single habitat type (e.g., alkaline grassland or bushland) whereas transects in TNP often cut across more heterogeneous habitats, therefore increasing the likelihood of encountering more mammal species per transect. However, walking transects may facilitate detecting species that are possibly more difficult to encounter and detect while driving transects. Either way, the “true” mammal species richness in both BWMA and TNP may be substantially higher, because species richness estimates were derived using diurnal transects and because we combined multiple species (e.g., mongoose species) (Steinbeiser, Kioko, Maresi, Kaitilia, & Kiffner, 2019). Indeed, recent camera trapping efforts in BWMA revealed the presence of multiple mammal species (e.g., aardvark *Orycteropus afer*, crested porcupine *Hystrix cristata*, honeybadger *Mellivora capensis*, striped hyena *Hyaena hyaena*) that were not detected during the transect counts (Kissui, Lobora, & Tosi, 2019). To more thoroughly compare species richness across sites, we thus recommend systematic camera trapping in both areas and utilizing a multispecies occupancy framework to quantify species richness while explicitly accounting for species‐ and area‐specific detection probabilities (Mc New & Handel, 2015).

While the persistence and positive population trends of multiple wildlife species can be interpreted as the ecological success of community‐based conservation efforts (Lee, 2018; Lee & Bond, 2018), the proliferation of wildlife populations may have negative repercussions for people adjacent to wildlife areas (Salerno et al., 2016). For example, it is possible that the increase in the BWMA elephant population may cause an upsurge of human–elephant conflict in the area. Indeed, elephants are frequently perceived as a considerable threat to crops, human safety, and property in the area (Bencin, Kioko, & Kiffner, 2016). In cooperation with multiple nongovernmental organizations, the management of BWMA addresses this issue by testing and implementing multiple strategies to prevent and mitigate crop damages by elephants (Chang'a et al., 2016; Hahn et al., 2017). Although benefit sharing models and adaptive management practices to curb damages by wildlife species and benefit sharing models are in place, these approaches could potentially be more effective (Brehony et al., 2018; Kicheleri, Treue, Nielsen, Kajembe, & Mombo, 2018), so that the ecological success of this community‐based conservation area does not come at the expense of local livelihoods (Salerno et al., 2016).

### 4.2 Measuring conservation effectiveness

Similar to other ecosystems (Mihoub et al., 2017), wildlife populations in the Tarangire‐Manyara ecosystem have fluctuated considerably over the long term in response to variation in anthropogenic and natural factors (Foley & Faust, 2010; Kiffner et al., 2017; Morrison et al., 2016). Thus, measuring the conservation effectiveness of protected areas requires time‐matched wildlife population data from suitable spatial reference points. Whether to choose areas with little or no conservation efforts as reference points (Lee, 2018; Lee & Bond, 2018), or fully protected national parks (Arcese & Sinclair, 1997; Sinclair & Dobson, 2015), may be a philosophical question. However, comparisons to fully protected areas can potentially yield stronger arguments for the conservation value and ecological integrity of community‐based conservation models.

Presently, most wildlife population monitoring schemes in Tanzania are carried out by aerial surveys (Stoner et al., 2007). Due to limited detection capabilities for most wildlife species, and relatively high costs, this monitoring technique is likely not ideal for assessing wildlife population parameters in community‐based conservation models (Caro, 2011; Greene, Bell, Kioko, & Kiffner, 2017; Jachmann, 2002; Lee & Bond, 2016). In addition to providing information on wildlife populations, participatory and ground‐based monitoring approaches (Danielsen et al., 2005; Msoffe et al., 2010; Schuette et al., 2018) may serve as an useful management tool for community‐based conservation models. Ground‐based wildlife monitoring yields other vital management data, such as detecting illegal activities. For example, two independent cases of illegal tree cutting were detected during the 2014 survey. Moreover, empowering WMA employees to monitor wildlife resources could be a crucial step toward decentralizing wildlife management in Tanzania (Benjaminsen et al., 2013; Kiwango et al., 2015). Ideally, wildlife monitoring schemes produce precise and unbiased density estimates (Yoccoz et al., 2001). In this case study, variation in encounter rates was the single most important factor contributing to uncertainty associated with density estimates (Appendix S6). To increase the precision of density estimates, employing a stratified sampling approach according to main habitat types may be a viable option (Barabesi & Fattorini, 2013). In cases where a (randomized or stratified) design‐based layout of transects is not possible, accounting for environmental and management related covariates may be a suitable option to further improve the robustness of results from wildlife monitoring (Oedekoven, Buckland, Mackenzie, Evans, & Burger, 2013; Schuette et al., 2018).

### 4.3 Conclusions and recommendations

Compared to a neighboring national park, we show that BWMA supports a similar mammal community and comparable species' densities. Most likely, BWMA provides complementary conservation functions that cannot be secured by national parks alone (including facilitating animal movement across seasonal ranges and providing permanent habitat for relatively rare species). However, given that BWMA constitutes only one example out of a total of 38 WMAs (22 with Authorized Association status) in Tanzania (CWMAC, 2019), participatory wildlife monitoring in multiple WMAs (ideally in relation to wildlife populations of adjacent national parks) is a crucial next step in measuring the general ecological effectiveness of this conservation model.

Despite the observed potential of BWMA to support a diverse and abundant mammal community, there are many challenges threatening the long‐term integrity of BWMA, including the rapidly growing human population, expansion of agriculture and human settlement, high frequency of livestock grazing in areas designated for wildlife, poaching, tree cutting, human‐wildlife conflicts, and power struggles among BWMA stakeholders (Bluwstein et al., 2016; Kicheleri et al., 2018; Kissui et al., 2019; Moyo et al., 2016). We recommend that the participatory management approach of BWMA need to be strengthened to ensure sustainable co‐existence between people and wildlife in Burunge.

## CONFLICT OF INTEREST

None declared.

## AUTHOR CONTRIBUTIONS

CK designed the study, executed field work, collated data, analyzed data and wrote the manuscript; ST executed field work, collated data, analyzed data and gave editorial advise; TS executed field work, collated data, analyzed data and gave editorial advise; VO'C executed field work, collated data, analyzed data and gave editorial advise; PS executed field work, collated data, analyzed data and gave editorial advise; JK designed the study, executed field work, collated data and gave editorial advise; BK designed the study, executed field work, collated data and gave editorial advise.

## Data Availability

Raw data of the transect observations are available at http://Dryad.org: https://doi.org/10.5061/dryad.w0vt4b8mf.
