# Supplementary material for: Community‐based wildlife management area supports similar mammal species richness and densities compared to a national park
Source: Ecol Evol. 2019 Dec 6;10(1):480–92. doi: 10.1002/ece3.5916 (PMC6972838; doi:10.1002/ece3.5916)
Supplement: Supplementary file 1 [file ECE3-10-480-s001.docx]

# Appendix

**S 1.** Species occurrence (X) in Burunge Wildlife Management Area and Tarangire National Park (TNP) assessed by line transect surveys conducted during the long rain, dry, and short rain seasons. Species are ordered in descending body mass. We estimated densities for species highlighted in bold.

|  | BWMA | | | TNP | | |
| --- | --- | --- | --- | --- | --- | --- |
| Species | Long rain | Dry | Short rain | Long rain | Dry | Short rain |
| Elephant  *Loxodonta africana* | **X** | **X** | **X** | **X** | **X** | **X** |
| Common hippopotamus  *Hippopotamus amphibius* |  |  | X |  |  |  |
| Giraffe  *Giraffa camelopardalis* | **X** | **X** | **X** | **X** | **X** | **X** |
| Eland  *Tragelaphus oryx* | X |  | X |  | X | X |
| Buffalo  *Syncerus caffer* | **X** | **X** | **X** | **X** | **X** | **X** |
| Zebra  *Equus quagga* | **X** | **X** | **X** | **X** | **X** | **X** |
| Wildebeest  *Connochaetes taurinus* | **X** | **X** | **X** |  | **X** | **X** |
| Lion  *Panthera leo* |  | X | X |  |  | X |
| Waterbuck  *Kobus ellipsiprymnus* | **X** | **X** | **X** | **X** | **X** | **X** |
| Coke’s hartebeest  *Alcelaphus buselaphus* | X |  | X | X |  | X |
| Warthog  *Phacochoerus africanus* | **X** | **X** | **X** | **X** | **X** | **X** |
| Lesser kudu  *Tragelaphus imberbis* | X |  | X |  | X | X |
| Spotted hyena  *Crocuta crocuta* | X |  | X |  |  |  |
| Grant’s gazelle  *Nanger granti* | X | X |  | X | X | X |
| Impala  *Aepyceros melampus* | **X** | **X** | **X** | **X** | **X** | **X** |
| Leopard  *Panthera pardus* |  |  | X |  |  |  |
| Bushbuck  *Tragelaphus scriptus* | X | X | X | X | X |  |
| Bohor reedbuck  *Redunca redunca* | X |  | X | X |  |  |
| Olive baboon  *Papio anubis* | X | X | X | X | X | X |
| Thomson’s gazelle  *Eudorcas thomsonii* | X | X | X | X |  | X |
| Bush duiker  *Sylvicapra grimmia* | X |  |  |  |  | X |
| Klipspringer  *Oreotragus oreotragus* |  |  |  |  |  | X |
| Steenbuck  *Raphicerus campestris* |  |  | X |  | X | X |
| Black-backed jackal  *Canis mesomelas* | X | X | X |  | X | X |
| Kirk’s dik-dik  *Madoqua kirkii* | **X** | **X** | **X** | **X** | **X** | **X** |
| Vervet Monkey  *Chlorocebus pygerythrus* | **X** | **X** | **X** | **X** | **X** | **X** |
| African wild cat  *Felis lybica* |  |  | X |  |  |  |
| Hare  *Lepus sp.* | X |  | X |  |  |  |
| Genet  *Genetta genetta / G. tigrina* |  |  | X |  |  |  |
| Bush hyrax  *Heterohyrax brucei* |  |  |  | X | X | X |
| Mongoose species  *Helogale sp., Herpestes sp.,*  *& Mungos sp.* | X |  |  | X | X | X |

**S 2.** (a) Model selection table and (b) regression coefficient estimates and associated standard errors, z- and p-values of the model averaged model (models within 2 AICc scores) to explain species richness at the transect level in Burunge Wildlife Management Area (BWMA) and Tarangire National Park. Abbreviations for the seasons are: LR=long rains, SR=short rains, dry=dry season.

| Model # | Intercept | Area | Transect length | Season | df | log-likelihood | AICc | Delta AICc | Model weight |
| --- | --- | --- | --- | --- | --- | --- | --- | --- | --- |
| 8 | 0.89 | + | 0.04 | + | 8 | -995.37 | 2007.10 | 0.00 | 0.64 |
| 6 | 1.04 | + |  | + | 7 | -997.16 | 2008.60 | 1.51 | 0.30 |
| 5 | 1.12 |  |  | + | 6 | -1000.27 | 2012.70 | 5.67 | 0.04 |
| 7 | 1.10 |  | 0.01 | + | 7 | -1000.19 | 2014.60 | 7.57 | 0.02 |
| 4 | 0.87 | + | 0.04 |  | 3 | -1024.80 | 2055.70 | 48.60 | 0.00 |
| 2 | 1.03 | + |  |  | 2 | -1026.96 | 2057.90 | 50.89 | 0.00 |
| 1 | 1.09 |  |  |  | 1 | -1029.11 | 2060.20 | 53.18 | 0.00 |
| 3 | 1.05 |  | 0.01 |  | 2 | -1028.80 | 2061.60 | 54.57 | 0.00 |
|  |  |  |  |  |  |  |  |  |  |
|  | Estimate | Std. Error | z-value | p-value |  |  |  |  |  |
| Intercept | 0.941 | 0.124 | 7.595 | <0.001 |  |  |  |  |  |
| Area (TNP vs. BWMA) | 0.178 | 0.068 | 2.622 | 0.009 |  |  |  |  |  |
| Transect length | 0.028 | 0.026 | 1.067 | 0.286 |  |  |  |  |  |
| Season (2012LR vs 2011SR) | -0.189 | 0.107 | 1.766 | 0.077 |  |  |  |  |  |
| Season (2014SR vs. 2011SR) | 0.064 | 0.095 | 0.671 | 0.502 |  |  |  |  |  |
| Season (2016LR vs. 2011SR) | -0.408 | 0.102 | 3.970 | <0.001 |  |  |  |  |  |
| Season (2016SR vs. 2011SR) | 0.252 | 0.090 | 2.774 | 0.006 |  |  |  |  |  |
| Season (2018Dry vs. 2011SR) | 0.033 | 0.094 | 0.346 | 0.729 |  |  |  |  |  |

**S 3.** Parameters associated with selected detection models (cds-hn: conventional distance sampling with half-normal key function; mcds-hn: multiple-covariate distance sampling with half normal key function) used to model species-specific wildlife densities in Burunge Wildlife Management Area (BWMA) and Tarangire National Park (TNP). ‘N’ indicates the number of detections, ‘P_a_’ the estimated detection probability, ‘P_a_ 95% CI’ the associated 95%-confidence interval, ‘ESW’ is the estimated strip width, and ‘chi^2^ gof-p’ is the p-value of the chi-squared goodness of fit-test.

| Species | Area | N | P_a_ | P_a_ 95% CI | ESW (m) | Model | chi^2^ gof-p |
| --- | --- | --- | --- | --- | --- | --- | --- |
| Elephant | BWMA | 43 | 0.463 | 0.321 - 0.668 | 170 | cds-hn | 0.372 |
| Elephant | TNP | 112 | 0.700 | 0.594 - 0.824 | 218 | cds-hn | 0.617 |
| Giraffe | BWMA | 94 | 0.795 | 0.655 - 0.965 | 165 | cds-hn | 0.817 |
| Giraffe | TNP | 73 | 0.603 | 0.500 - 0.726 | 178 | cds-hn | 0.632 |
| Buffalo | BWMA | 24 | 0.425 | 0.293 - 0.617 | 106 | mcds-hn | 0.438 |
| Buffalo | TNP | 21 | 0.616 | 0.445 - 0.853 | 246 | cds-hn | 0.624 |
| Zebra | BWMA | 226 | 0.634 | 0.567 - 0.710 | 185 | cds-hn | 0.455 |
| Zebra | TNP | 228 | 0.475 | 0.403 - 0.561 | 103 | cds-hn | 0.117 |
| Wildebeest | BWMA | 148 | 0.679 | 0.584 - 0.789 | 204 | cds-hn | 0.040 |
| Wildebeest | TNP | 80 | 0.606 | 0.504 - 0.729 | 150 | cds-hn | 0.573 |
| Waterbuck | BWMA | 20 | 0.432 | 0.237 - 0.788 | 128 | cds-hn | 0.853 |
| Waterbuck | TNP | 56 | 0.458 | 0.388 - 0.541 | 117 | cds-hn | 0.220 |
| Warthog | BWMA | 65 | 0.562 | 0.462 - 0.683 | 95 | cds-hn | 0.212 |
| Warthog | TNP | 113 | 0.587 | 0.514 - 0.671 | 82 | mcds-hn | 0.012 |
| Impala | BWMA | 118 | 0.474 | 0.380 - 0.592 | 62 | cds-hn | 0.069 |
| Impala | TNP | 237 | 0.595 | 0.547 - 0.647 | 106 | mcds-hn | 0.344 |
| Kirk’s dik-dik | BWMA | 138 | 0.401 | 0.321 - 0.501 | 25 | cds-hn | 0.117 |
| Kirk’s dik-dik | TNP | 88 | 0.739 | 0.642 - 0.850 | 36 | mcds-hn | 0.556 |
| Vervet Monkey | BWMA | 58 | 0.598 | 0.485 - 0.737 | 67 | cds-hn | 0.697 |
| Vervet Monkey | TNP | 41 | 0.514 | 0.340 - 0.777 | 44 | cds-hn | 0.864 |

**S 4.** Season- and species specific density estimates (D) incl. 95% confidence intervals) and results of pairwise density comparisons between Burunge Wildlife Management Area and Tarangire National Park assessed via a z-test. Due to multiple testing, p-values were Bonferroni corrected. Significant differences (p<0.05) were highlighted in bold. Abbreviations for the seasons are: LR=long rains, SR=short rains, dry=dry season.

| Species | Season | BWMA D (95% CI) | TND D (95% CI) | Z-score | p-value |
| --- | --- | --- | --- | --- | --- |
| Elephant | 2011SR | 0.18 (0.03-1.06) | 1.83 (0.86-3.89) | -2.234 | 0.155 |
| Elephant | **2012LR** | **0.30 (0.08-1.10)** | **9.65 (6.00-15.54)** | **-3.984** | **≤0.001** |
| Elephant | **2014SR** | **0.03 (0.00-0.14)** | **3.18 (1.56-6.47)** | **-2.702** | **0.042** |
| Elephant | 2016LR | 0.61 (0.11-3.34) | 2.54 (1.21-5.32) | -1.740 | 0.502 |
| Elephant | 2016SR | 1.25 (0.46-3.37) | 4.87 (2.83-8.38) | -2.404 | 0.098 |
| Elephant | 2018Dry | 2.29 (0.78-6.72) | 3.47 (1.74-6.92) | -0.648 | 1.000 |
| Giraffe | 2011SR | 2.72 (0.94-7.91) | 0.59 (0.25-1.38) | 1.365 | 1.000 |
| Giraffe | 2012LR | 1.13 (0.45-2.86) | 0.88 (0.25-3.06) | 0.321 | 1.000 |
| Giraffe | 2014SR | 2.13 (0.73-6.18) | 0.68 (0.35-1.35) | 0.675 | 1.000 |
| Giraffe | 2016LR | 1.27 (0.45-3.59) | 1.59 (0.62-4.10) | -0.301 | 1.000 |
| Giraffe | 2016SR | 0.62 (0.22-1.73) | 1.36 (0.70-2.66) | -0.969 | 1.000 |
| Giraffe | 2018Dry | 2.84 (1.41-5.69) | 2.35 (1.13-4.89) | 0.359 | 1.000 |
| Buffalo | 2011SR | 0.00 | 0.24 (0.07-0.84) | -1.488 | 0.833 |
| Buffalo | 2012LR | 0.00 | 0.00 | NA | NA |
| Buffalo | 2014SR | 0.57 (0.11-3.06) | 4.76 (0.77-29.54) | -0.841 | 1.000 |
| Buffalo | 2016LR | 0.29 (0.07-1.09) | 0.04 (0.01-0.21) | 1.200 | 1.000 |
| Buffalo | 2016SR | 2.84 (0.14-57.76) | 13.18 (3.45-50.43) | -1.026 | 1.000 |
| Buffalo | 2018Dry | 0.68 (0.22-2.12) | 0.03 (0.00-0.14) | 1.568 | 0.713 |
| Zebra | 2011SR | 15.22 (4.78-48.50) | 52.37 (30.23-90.71) | -2.122 | 0.204 |
| Zebra | 2012LR | 11.21 (6.02-20.88) | 2.42 (0.94-6.26) | 2.354 | 0.113 |
| Zebra | 2014SR | 16.09 (7.68-33.70) | 40.52 (25.51-64.36) | -2.143 | 0.194 |
| Zebra | **2016LR** | **11.23 (5.54-22.75)** | **0.00** | **2.751** | **0.036** |
| Zebra | **2016SR** | **15.32 (8.40-27.94)** | **46.77 (29.74-73.56)** | **-2.663** | **0.047** |
| Zebra | **2018Dry** | **6.76 (3.39-13.48)** | **101.94 (57.53-180.64))** | **-3.155** | **0.010** |
| Wildebeest | 2011SR | 4.39 (1.03-18.68) | 31.75 (15.00-67.20) | -2.131 | 0.199 |
| Wildebeest | 2012LR | 5.14 (1.62-16.35) | 0.00 | 1.661 | 0.581 |
| Wildebeest | 2014SR | 1.51 (0.63-3.57) | 24.25 (9.20-63.92) | -1.858 | 0.386 |
| Wildebeest | 2016LR | 16.52 (7.52-36.29) | 0.00 | 2.441 | 0.088 |
| Wildebeest | 2016SR | 37.99 (19.81-72.85) | 28.59 (11.46-71.33) | 0.502 | 1.000 |
| Wildebeest | 2018Dry | 56.60 (28.08-114.05) | 42.76 (19.13-95.59) | -1.376 | 1.024 |
| Waterbuck | 2011SR | 0.00 | 5.35 (2.36-12.12) | -2.360 | 0.110 |
| Waterbuck | 2012LR | 0.69 (0.18-2.67) | 1.07 (0.34-3.30) | -0.465 | 1.000 |
| Waterbuck | 2014SR | 0.57 (0.15-2.21) | 1.68 (0.74-3.82) | -1.334 | 1.000 |
| Waterbuck | 2016LR | 0.00 | 1.52 (0.69-3.33) | -2.436 | 0.091 |
| Waterbuck | 2016SR | 0.00 | 2.02 (0.69-5.91) | -1.781 | 0.450 |
| Waterbuck | 2018Dry | 0.62 (0.22-1.75) | 0.55 (0.10-3.01) | 0.116 | 1.000 |
| Warthog | 2011SR | 0.56 (0.18-1.73) | 2.49 (1.38-4.51) | -2.351 | 0.113 |
| Warthog | 2012LR | 0.39 (0.10-1.56) | 2.48 (0.70-8.79) | -1.212 | 1.358 |
| Warthog | 2014SR | 1.43 (0.44-4.70) | 4.37 (2.45-7.80) | -1.862 | 0.377 |
| Warthog | 2016LR | 1.36 (0.54-3.45) | 3.14 (1.24-7.95) | -1.059 | 1.000 |
| Warthog | 2016SR | 4.39 (2.01-9.59) | 4.81 (3.00-7.72) | -0.200 | 1.000 |
| Warthog | 2018Dry | 2.29 (1.07-4.90) | 5.21 (2.64-10.27) | -1.436 | 0.916 |
| Impala | 2011SR | 4.72 (1.08-20.59) | 11.18 (6.07-20.59) | -1.265 | 1.000 |
| Impala | 2012LR | 2.88 (0.82-10.10) | 34.44 (15.76-75.24) | -2.235 | 0.155 |
| Impala | 2014SR | 2.91 (1.37-6.19) | 10.81 (6.27-18.65) | -2.446 | 0.088 |
| Impala | 2016LR | 12.09 (5.13-28.47) | 21.51 (11.86-39.00) | -1.102 | 1.000 |
| Impala | 2016SR | 12.00 (5.31-27.16) | 19.19 (11.14-33.07) | -0.970 | 1.000 |
| Impala | 2018Dry | 33.78 (18.71-61.01) | 19.89 (9.42-42.01) | 1.077 | 1.000 |
| Kirk’s dik-dik | 2011SR | 1.60 (0.63-4.07) | 3.94 (2.18-7.12) | -1.650 | 0.594 |
| Kirk’s dik-dik | 2012LR | 4.06 (1.76-9.40) | 0.75 (0.14-3.97) | 1.761 | 0.470 |
| Kirk’s dik-dik | 2014SR | 3.89 (1.60-9.45) | 5.97 (3.21-11.09) | -0.797 | 1.000 |
| Kirk’s dik-dik | 2016LR | 2.49 (1.15-5.39) | 4.84 (2.29-10.23) | -1.107 | 1.000 |
| Kirk’s dik-dik | 2016SR | 7.18 (4.06-12.71) | 3.67 (2.07-6.52) | 1.492 | 0.817 |
| Kirk’s dik-dik | 2018Dry | 8.12 (4.33-15.23) | 1.17 (0.33-4.21) | 2.535 | 0.068 |
| Vervet monkey | 2011SR | 3.88 (1.11-13.55) | 4.34 (1.60-11.78) | -0.131 | 1.000 |
| Vervet monkey | 2012LR | 2.51 (1.00-6.30) | 4.83 (1.31-17.81) | -0.650 | 1.000 |
| Vervet monkey | 2014SR | 3.32 (0.85-12.94) | 4.25 (1.75-10.31) | -0.292 | 1.000 |
| Vervet monkey | 2016LR | 2.81 (0.61-13.04) | 3.63 (0.94-13.95) | -0.236 | 1.000 |
| Vervet monkey | 2016SR | 5.21 (1.65-16.44) | 2.71 (1.01-7.30) | 0.737 | 1.000 |
| Vervet monkey | 2018Dry | 9.29 (4.27-20.24) | 2.80 (0.89-8.83) | 1.567 | 0.713 |

**S 5.** Kendall’s correlation coefficient estimates (tau) and associated p-values, testing the temporal trend of wildlife populations in Burunge Wildlife Management Area (BWMA; n=7) and Tarangire National Park (TNP; n=6) from 2011 to 2018.

|  | **BWMA** | | **TNP** | |
| --- | --- | --- | --- | --- |
| **Species** | **tau** | **p-value** | **tau** | **p-value** |
| Elephant | **0.840** | **0.018** | -0.236 | 0.653 |
| Giraffe | -0.061 | 0.897 | **0.905** | **0.013** |
| Buffalo | 0.524 | 0.228 | 0.236 | 0.652 |
| Zebra | -0.113 | 0.810 | 0.435 | 0.389 |
| Wildebeest | **0.843** | **0.017** | 0.296 | 0.568 |
| Waterbuck | 0.166 | 0.722 | -0.661 | 0.153 |
| Warthog | 0.707 | 0.075 | **0.816** | **0.048** |
| Impala | **0.770** | **0.043** | 0.000 | 0.999 |
| Kirk’s dik-dik | 0.498 | 0.255 | -0.065 | 0.903 |
| Vervet monkey | 0.594 | 0.160 | **-0.871** | **0.024** |

**S 6.** Component percentages of variation in density estimates of ten wildlife species in Burunge Wildlife Management Area (BWMA) and Tarangire National Park (TNP) associated with detection probability, encounter rates, and group sizes.

|  | **BWMA** | | | |  | **TNP** | | |
| --- | --- | --- | --- | --- | --- | --- | --- | --- |
|  | **Detection probability** | **Encounter rate** | | **Group size** |  | **Detection probability** | **Encounter rate** | **Group size** |
| Elephant | 6.1 | 62.7 | 32.6 | |  | 6.7 | 63.6 | 29.7 |
| Giraffe | 4.0 | 53.7 | 42.3 | |  | 5.1 | 64.1 | 30.8 |
| Buffalo | 24.1 | 49.1 | 33.5 | |  | 3.5 | 65.7 | 51.3 |
| Zebra | 2.3 | 56.5 | 41.2 | |  | 9.2 | 52.1 | 38.7 |
| Wildebeest | 2.8 | 61.2 | 36.0 | |  | 4.5 | 52.6 | 43.0 |
| Waterbuck | 18.0 | 68.8 | 17.7 | |  | 2.8 | 60.9 | 36.3 |
| Warthog | 3.8 | 69.2 | 27.0 | |  | 21.2 | 62.7 | 16.1 |
| Impala | 5.7 | 46.3 | 48.0 | |  | 10.3 | 49.9 | 39.8 |
| Kirk’s dik-dik | 10.1 | 83.2 | 6.7 | |  | 20.7 | 70.1 | 11.1 |
| Vervet monkey | 3.7 | 56.7 | 39.6 | |  | 12.9 | 67.2 | 19.9 |
|  |  |  |  | |  |  |  |  |
| Average | 8.0 | 60.7 | 32.5 | |  | 9.7 | 60.9 | 31.7 |
